# Cytokine profile in childhood-onset systemic lupus erythematosus: a cross-sectional and longitudinal study

**DOI:** 10.1590/1414-431X20175738

**Published:** 2017-04-03

**Authors:** A. Cavalcanti, R. Santos, Z. Mesquita, A.L.B.P. Duarte, N. Lucena-Silva

**Affiliations:** 1 Unidade de Reumatologia, Hospital das Clínicas, Universidade Federal de Pernambuco, Recife, PE, Brasil; 2 Departamento de Imunologia, Centro de Pesquisas Aggeu Magalhães, Fundação Oswaldo Cruz, Recife, PE, Brasil; 3 Unidade de Reumatologia Pediátrica, Instituto de Medicina Integral Professor Fernando Figueira, Recife, PE, Brasil; 4 Unidade de Oncologia Pediátrica, Instituto de Medicina Integral Professor Fernando Figueira, Recife, PE, Brasil

**Keywords:** Cytokines, Childhood-onset systemic lupus erythematosus, Disease activity, SLEDAI-2K, Inflammation

## Abstract

Childhood-onset systemic lupus erythematosus (cSLE) exhibits an aggressive clinical phenotype and severe complications. This could be due to a pro-inflammatory cytokine milieu. Therefore, we determined plasma levels of Th1 (IL-2, IFN-γ, TNF), Th2 (IL-4), Th17 (IL-17A, IL-6), and Treg (IL-10) cytokines in a cohort of cSLE patients and healthy controls, and we evaluated the association between these cytokines and disease activity. We conducted a cross-sectional study with 51 cSLE patients from two pediatric rheumatology services. Ten cSLE patients participated in a longitudinal follow-up study. Blood samples were collected from the same patient during active and inactive disease. Disease activity was evaluated according to SLE Disease Activity Index 2000 (SLEDAI-2K). Cytokines levels were measured by cytometric bead array technique. cSLE patients had higher IL-6 (P<0.001) and IL-10 (P<0.001) levels than healthy controls. Patients with active disease had higher IL-6 and IL-10 levels than patients with inactive disease (P=0.001 and P=0.014, respectively) and the control group (both P<0.001). IL-6 (P=0.022), IL-10 (P=0.013), and IL-17A (P=0.041) levels were significantly higher during active than inactive disease. Linear regression analysis revealed IL-6 (P=0.002, 95%CI=0.006-0.025) and IL-10 (P=0.01 95%CI=0.021-0.150) as independent factors for increased SLEDAI-2K. IL-6, IL-10, and IL-17A are candidate biomarkers for disease activity in cSLE patients. This is the first longitudinal study to support their pivotal role in the pathogenesis of the disease.

## Introduction

Childhood-onset systemic lupus erythematosus (cSLE; diagnosis at age younger than 18 years) affects approximately 15–20% of all SLE patients (1). Like SLE in adults, cSLE is a ubiquitous disease that has been described in different ethnic groups, with higher prevalence and severity in non-Caucasian populations (2). However, studies that compared cohorts of adult-onset SLE and cSLE patients demonstrated higher disease activity and severity in the latter group, mainly due to renal and neuropsychiatric manifestations (3). Furthermore, children and adolescents need more glucocorticoids and immunosuppressive therapy than adults, present higher mortality rates, and higher treatment costs (3).

The pathogenesis of SLE is complex and multifactorial; it involves environmental, hormonal, and immunological factors in a genetically predisposed individual. Both innate and adaptive immunity contribute to tissue damage. Dysregulation of innate immunity includes a high expression level of interferon (IFN)-α, activation of dendritic cells, impaired phagocytosis, complement consumption, and increased production of neutrophil extracellular traps (4). However, adaptive immunity is augmented with excessive autoantibody production due to abnormalities in B and T lymphocytes (4). Cytokines have an essential role in controlling the differentiation, maturation, and activation of the different immune cell types previously described. The final consequence of the immune system dysregulation is local inflammation in a target tissue with subsequent tissue damage. Therefore, cytokines have an important role in the pathogenesis of SLE and are possible therapeutic targets and disease biomarkers because their levels vary with disease activity (5).

T helper cells (Th) are characterized by the different cytokine profiles they produce (6). The Th1 profile mainly produces interleukin (IL)-2, IFN-γ, and tumor necrosis factor (TNF). However, IL-4, IL-5, and IL-13 are part of the Th2 profile. The Th17 profile is mainly characterized by IL-17A and IL-6, whereas regulatory Th cells (Treg) produce the anti-inflammatory cytokines IL-10 and transforming growth factor-β. Regardless of the patient's age at SLE onset, cytokines play an important role in disease pathogenesis. Studies have shown different cytokine profiles for Th1, Th2, Th17, and Treg cells in adult SLE patients compared to a healthy control group, as well as differences in disease phenotype and activity (7–12). However, only a few studies have investigated the cytokine profile of cSLE (13–16). Therefore, we conducted a cross-sectional and longitudinal study to determine the profiles of IL-2, IL-4, IL-6, IL-10, IL-17A, IFN-γ, and TNF cytokines in cSLE patients and a healthy control group, and evaluated the association of these cytokines with disease activity.

## Material and Methods

### Patients and controls

Fifty-one consecutive cSLE patients were recruited from the Hospital das Clínicas, Universidade Federal de Pernambuco (n=32) and the Unidade de Reumatologia Pediátrica, Instituto de Medicina Integral Professor Fernando Figueira (n=19). This study was carried out between June 2012 and July 2015. To be included in the study, patients were required to: i) fulfill at least four SLE classification criteria of the American College of Rheumatology (ACR) (17); ii) be younger than 18 years of age at disease diagnosis; and iii) have participated in at least 6 months of follow-up in their respective clinics. A healthy control group with 47 children and adolescents from municipal schools paired by gender and age with the cSLE patients was also included in the study. These children were evaluated in the presence of their parent or legal guardian, and only those with no family history of autoimmune diseases, allergies, or cancer were selected for the study. On the day of blood collection, all participants in both groups (cSLE patients and healthy control subjects) underwent medical evaluation by authors AC or ZM to rule out any possibility of infection.

Of the 51 cSLE patients, 10 participated in a longitudinal follow-up study in which blood samples were collected from the same patient at two distinct time points: when the disease was active and when it was inactive (or vice versa). Disease activity was evaluated following the parameters of the Systemic Lupus Erythematosus Disease Activity Index 2000 (SLEDAI-2K) (18).

The Ethics Committee of both institutions approved the present study. Data collection was performed after obtaining written informed consent signed by the participants' parent or legal guardian.

### Demographic, clinical, and laboratory manifestations and disease activity

The demographic and laboratory data of all patients were recorded on the day of blood collection, as was the treatment prescribed for cSLE during the past 3 months. Data included patient current age, age at disease onset (age when he/she developed the first symptoms solely attributed to cSLE), and disease duration (period elapsed from disease onset until blood collection). Age at disease diagnosis was not considered, since it was similar to the age at disease onset.

Nephritis was diagnosed based on proteinuria level >0.5 g/24 h with active urinary sediment and/or renal biopsy results consistent with lupus nephritis. Hematological manifestations were defined as thrombocytopenia (<150,000 platelets/mm^3^), leukopenia (<3,000 leukocytes/mm^3^), and autoimmune hemolytic anemia after exclusion of infection or any drug use. Malar rash, discoid rash, photosensitivity, cutaneous vasculitis, and oral ulcers were characterized as mucocutaneous manifestations. Neuropsychiatric, serositis and arthritis were also considered clinical manifestations of SLE.

Anti-double stranded DNA (anti-dsDNA) antibodies were determined by indirect immunofluorescence using *Crithidia* as substrate and were considered positive if they were higher than 1:10. The C3 and C4 serum levels were determined by nephelometry.

Disease activity was assessed by the SLEDAI-2K score in the past 10 days, and scores ≥4 were considered to be active disease (19). We also measured cumulative damage in our patients by the Systemic Lupus International Collaborating Clinics (SLICC)/ACR Damage Index (SDI). Damage was considered present if scores were ≥1 (20).

### Cytokine assay

Blood samples were collected, plasma was separated by density gradient centrifugation using the Ficoll-Paque^®^ reagent (GE Healthcare, USA), and aliquots were stored at −80°C until analysis. Levels of IL-2, IL-4, IL-6, IL-10, IL-17A, IFN-γ, and TNF cytokines were determined using the Cytometric Bead Array Human Th1/Th2/Th17 Cytokine Kit (BD Biosciences, USA). Quantification was performed using the BD Accuri^™^ C6 flow cytometer (BD Biosciences) and the results were analyzed using FCAPArray^™^ software (BD Biosciences). The experiment was performed according to the manufacturer's protocol and the results are reported in pg/mL. The detection limits for IL-2, IL-4, IL-6, IL-10, IL-17A, IFN-γ, and TNF were 2.6, 4.9, 2.4, 18.9, 3.8, 3.7, and 4.5 pg/mL, respectively.

### Statistical analysis

Statistical analyses were performed using SPSS Statistics software version 20 (USA). Continuous variables are reported as mean±SD or median and range, and categorical variables are reported as absolute values and percentages. The differences in cytokine levels between the studied groups were examined by the Kruskal-Wallis non-parametric test followed by the Mann-Whitney U-test. The Wilcoxon test was used to assess the differences in cytokines between the patients in the longitudinal follow-up study. Spearman rank correlation was used to correlate cytokine levels (independent variable) with SLEDAI-2K score. Then, only independent variables with P<0.20 were considered for multivariate analysis. Multivariate analysis was performed using a linear regression model (backward stepwise) to understand the association between all the cytokines and the logarithm of the SLEDAI-2K variable. P<0.05 was considered to be statistically significant.

## Results

### Demographic, clinical, laboratory, and treatment data

The studied groups (cSLE patients and healthy control subjects) were homogeneous for gender and age. Of the 51 cSLE patients, 47 (92%) were female and the median age was 15 years (range 5–20). The median disease duration was 3 years (range 1–9). The median time between the first and second blood collection in the longitudinal study was 11 months (range 9–13). Of the 47 healthy control subjects, 43 (91%) were female and the median age was 15 years (range 6–21).

At blood collection, 26 patients presented with active disease (SLEDAI-2K ≥4), and a median score of 6 (range 4–27), and 25 patients presented with inactive disease, and a median score of 2 (range 0–2). Active nephritis (21%), mucocutaneous disorders (16%), and arthritis (12%) were the most common clinical manifestations. All patients were using hydroxychloroquine on the day of blood collection, and 31 (61%) were using prednisone, 25 (49%) were using mycophenolate mofetil, 17 (33%) were using azathioprine, and only 4 (8%) were using methotrexate (Table 1). When indicated, intravenous high dose methylprednisolone was administered after blood collection.

**Table:**
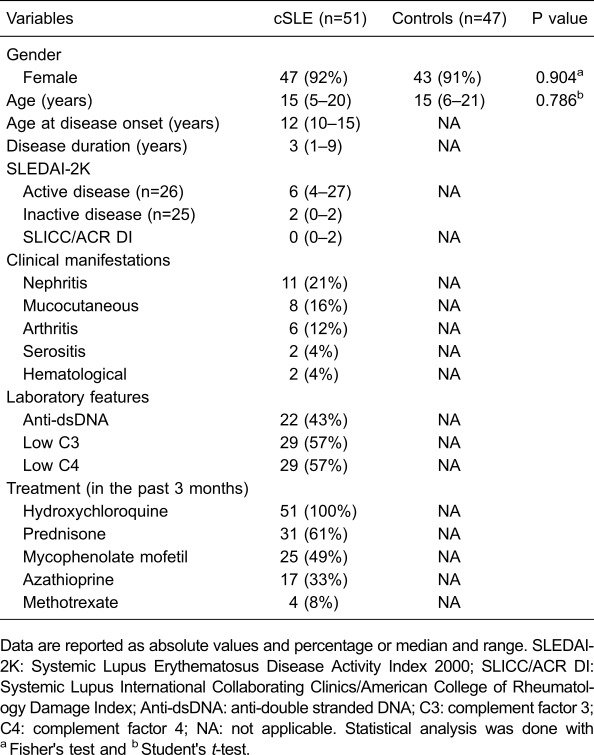

### Cytokine secretion profile

Plasma levels of the Th1, Th2, Th17, and Treg cytokines of cSLE patients and healthy control subjects are shown in Table 2. The plasma levels of IL-6 (P<0.001) and IL-10 (P<0.001) were significantly higher in cSLE patients than in the healthy control subjects. No significant difference was observed between the studied groups for IL-2, IL-4, IL-17A, IFN-γ, and TNF cytokines.

**Table:**
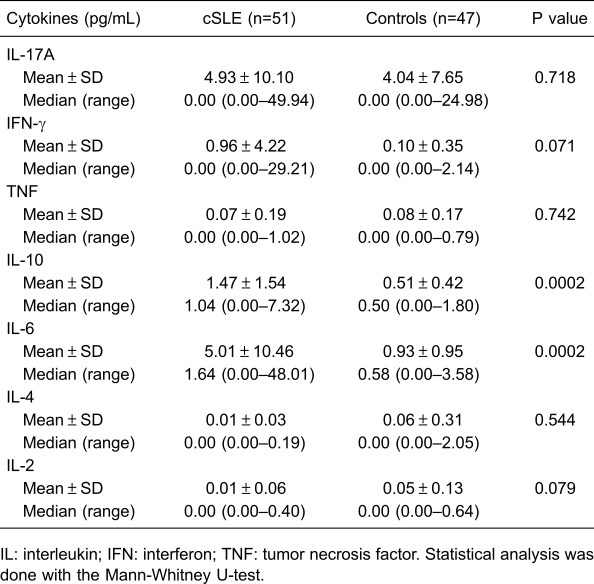

**Figure 1:**
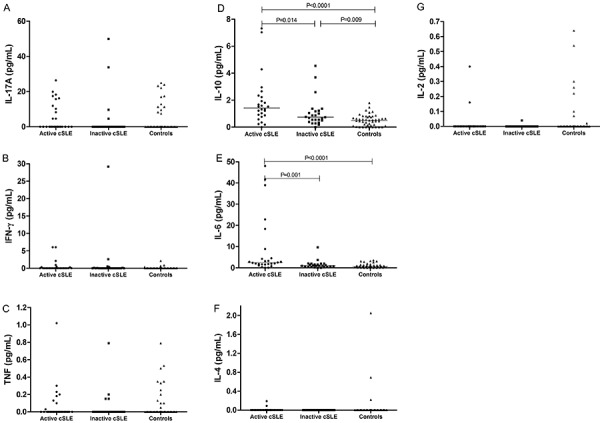
Th1, Th2, Th17, and Treg profiles of patients with active and inactive childhood-onset systemic lupus erythematosus and healthy controls. IL: interleukin; IFN: interferon; TNF: tumor necrosis factor. Data are reported as absolute values and the vertical line represents the median. Statistical analysis was done with the Kruskal-Wallis test followed by the Mann-Whitney U-test.

The plasma levels of IL-10 were higher in patients with active disease than in patients with inactive disease (P=0.014) and healthy control subjects (P<0.001). Patients with inactive disease also showed higher IL-10 levels than the healthy control group (P=0.009). Furthermore, patients with active disease had significantly higher levels of IL-6 than patients with inactive disease (P=0.001) and healthy control subjects (P<0.001). However, no difference was observed between patients with inactive disease and the healthy control group regarding IL-6 level. No significant difference was observed between patients with active or inactive disease and the healthy control group for the other cytokines studied (Figure 1).

In the longitudinal follow-up study in which blood samples were collected from 10 patients at two distinct moments (active and inactive disease or vice versa), significantly higher levels of IL-10 (P=0.013), IL-6 (P=0.022), and IL-17A (P=0.041) were observed during active disease when compared with inactive disease (Table 3).

**Table:**
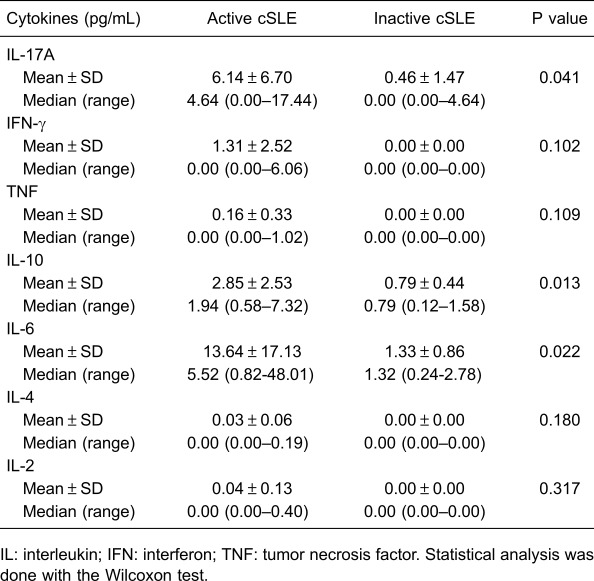

Table 4 presents the correlations of the cytokines with the SLEDAI-2K score. To detect independent associations of the cytokines with SLEDAI-2K, linear regression was conducted only with important cytokines (P<0.20). In the final model, IL-10 [P=0.01; 95% confidence interval (CI)=0.021-0.150] and IL-6 (P=0.002; 95%CI=0.006–0.025) were confirmed as independent factors for an increase in the SLEDAI-2K score.

**Table:**
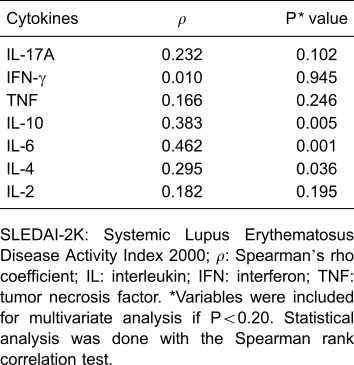

No statistically significant association was found for the plasma levels of the different cytokines evaluated and the treatments prescribed in the patient population (data not shown).

Of the 51 patients, only 6 presented some kind of damage (2 cataract, 2 avascular necrosis, 1 renal failure and 1 cognitive deficit), and only one patient presented a score of 2 (bilateral avascular necrosis). Also, no association between cytokines levels and cumulative damage (SDI score) was observed.

## Discussion

SLE is the prototype of autoimmune diseases, and its pathogenesis is highly affected by innate and adaptive immunity. Although SLE is mainly characterized by deposition of immune complexes and autoantibody production, cytokines also have an important role in immune system dysregulation in SLE because they act on the differentiation, maturation, and activation of several effector cells, culminating in inflammation and subsequent tissue damage. While there are several studies of cytokine profiles in adult SLE patients (7–12), studies of cytokines in cSLE are incipient (13–16).

In our study, we found increased levels of IL-10 in cSLE patients and an association of this cytokine with disease activity in both the cross-sectional and the longitudinal follow-up studies, as well as in the multivariate analysis. In a Thai population, high serum levels of IL-10 and association with disease activity were reported in cSLE patients (21). In a population in southern Brazil, increased levels of IL-10 were associated with anti-dsDNA in cSLE (14 [Bibr B15]). The majority of studies involving adult SLE patients also reported increased levels of IL-10, which were associated with disease activity and increased production of anti-dsDNA (22). IL-10 is the most important anti-inflammatory cytokine (23). Unlike patients with other autoimmune diseases such as psoriasis and rheumatoid arthritis, for which IL-10 levels are low, SLE patients present high levels of IL-10 (24). IL-10 increases the proliferation and differentiation of B lymphocytes and induces the production of autoantibodies by these cells in SLE (24). In SLE animal models, continuous administration of anti-IL-10 antibody to NZB/W F1 mice delayed the onset of autoimmunity (25). Moreover, a small series with six SLE patients showed that administration of anti-IL-10 monoclonal antibody caused improvement in cutaneous lesions, joint symptoms, and the SLEDAI index (26). Taken together, these results, although preliminary, suggest a possible therapeutic effect of anti-IL-10 in SLE patients.

In the present study, increased levels of IL-6 were observed in cSLE patients and were associated with disease activity (in the cross-sectional and longitudinal study) and SLEDAI-2K. Only one study has described increased levels of IL-6 in cSLE patients, and it found no association of this cytokine with disease activity (14). IL-6 is a classical pro-inflammatory cytokine produced by a variety of cells. It has several biological functions; the most important for SLE is the final stage of maturation of B lymphocytes into plasma cells and the consequent greater production of immunoglobulins (27). Studies involving adult SLE patients have indicated high levels of IL-6 and its association with disease activity and anti-dsDNA (8,12). In addition to the systemic effects, IL-6 may act locally as demonstrated by the presence of IL-6 in the cerebrospinal fluid in patients with neuropsychiatric involvement (28), and in urine in patients with nephritis (29). Furthermore, in SLE animal models, exogenous administration of recombinant human IL-6 in NZB/W mice accelerated the development of glomerulonephritis and increased mortality (30).

There were no differences in IL-17A plasma levels in cSLE patients compared to healthy children in the cross-sectional study. However, in the longitudinal follow-up study of patients at two different time points, there was a significant increase in IL-17A during active disease that corroborates with the data of previous studies (16,31). Although the longitudinal follow-up was conducted with a small number of patients, this study design allowed us to demonstrate a stronger association between IL-17A and disease activity than would a cross-sectional design, in part due to the patients being their own control. In SLE, IL-17A promotes inflammation by acting in both innate and adaptive immunity. It causes tissue damage through the recruitment of neutrophils and macrophages and facilitates infiltration of T cells. IL-17A also stimulates the proliferation of B lymphocytes and autoantibody production and inhibits differentiation of Treg cells (6). The primary cells that produce IL-17A are Th17 lymphocytes and CD3^+^CD4^-^CD8^-^ double-negative (DN) T cells (32). In adult SLE patients, both the IL-17A and Th17 cells showed a positive association with the SLEDAI index (32,33). Recently, Th17 cells and DN T cells have been encountered in renal biopsy specimens of patients with lupus nephritis (32). Studies in cSLE have also presented an increased level of IL-17A and its correlation with SLEDAI (16), as well as the predominance of Th17 cells and their association with active lupus nephritis (31).

The relationship between TNF and SLE remains uncertain (5). The results are controversial in animal models and in humans; depending on the animal model used, the results point to opposite conclusions. In NZB/W mice, for example, lower production of TNF was associated with autoimmune manifestations (34). However, in MRL/lpr mice, an increased expression of TNF was observed in the serum and in the kidney tissue (35). Human studies involving adult patients showed increased serum levels of TNF with (10,11) or without (36) association with disease activity. Another study presented high levels of TNF in patients with inactive disease, suggesting a protective effect of this cytokine in SLE (9).

Studies involving cSLE patients also showed contradictory results for TNF. Rana et al. (13) found high gene expression (mRNA) of TNF in peripheral blood of cSLE patients; however, no significant difference was observed in the serum levels of TNF in patients and controls. However, Postal et al. (14) demonstrated increased levels of TNF and association with disease activity in cSLE patients. In our study, we observed no difference in the TNF levels of patients and healthy control subjects, and a lack of association with SLEDAI-2K score. The differences between studies might be explained by the fact that patients were using hydroxychloroquine, especially in our study, in which all patients were using this anti-malarial drug that inhibits the production of TNF (37). The low level of TNF observed in our study may also justify the low level of IFN-γ, because TNF leads to increased production of IFN-γ (38).

The lack of correlation between cytokine levels and SLICC/ACR damage index may be explained by the fact that few children presented cumulative damage. Furthermore, damage such as cataract and vascular necrosis may be a consequence of therapy adverse effects and not related to cumulative disease activity (39).

An important limitation of our study was the small number of patients. To reduce the effects of this limitation, we recruited the majority of cSLE patients from the only two reference centers for pediatric rheumatology in our region (Pernambuco State, Northeast, Brazil).

In conclusion, our results, especially those of the longitudinal follow-up study, support the pivotal roles of IL-10, IL-6, and IL-17A in the pathogenesis of cSLE. To our knowledge, this is the first longitudinal follow-up study involving SLE patients from a pediatric population analyzing these cytokines. IL-6, IL-10, and IL-17A are important candidates for biomarkers of disease activity and targets of anti-cytokine therapy for the treatment of SLE. Before their use in clinical practice, the appropriate cut-off levels of these cytokines need to be established in order to differentiate the active and inactive phases of the disease (40). Our results provide important knowledge for future research, but longitudinal studies with a larger number of patients should be performed for a better comprehension of cytokines balance and disease activity.
